# A Mobile Phone App Designed to Support Weight Loss Maintenance and Well-Being (MotiMate): Randomized Controlled Trial

**DOI:** 10.2196/12882

**Published:** 2019-09-04

**Authors:** Emily Brindal, Gilly A Hendrie, Jill Freyne, Manny Noakes

**Affiliations:** 1 Health and Biosecurity Commonwealth Scientific and Industrial Research Organisation Adelaide Australia; 2 Health and Biosecurity Commonwealth Scientific and Industrial Research Organisation Epping Australia

**Keywords:** mobile phone, body weight, lifestyle, mood, happiness

## Abstract

**Background:**

Few people successfully maintain lost weight over the longer term. Mobile phones have the potential to deliver weight loss management programs that can encourage self-monitoring while also providing some behavioral therapy to assist users in developing personal skills that may be necessary for improved longer term weight loss maintenance.

**Objective:**

The aim of this study was to evaluate a program supporting weight maintenance, which uses a behaviorally based mobile phone app to manage weight, food, exercise, mood, and stress.

**Methods:**

In a randomized controlled trial over 24 weeks, the full version of the app (MotiMate) was compared with a control app (monitoring only; excluding mood and stress) for its effect on weight, diet, and psychological well-being. Both apps had the same visual appearance and were designed to deliver all intervention content without face-to-face contact. The control version included features to track weight, food intake, and exercise with limited feedback and no encouraging/persuasive features. The intervention app included more persuasive and interactive features to help users track their weight, food intake, and physical activity and prompted users to enter data each day through notifications and included a mood and stress workshopping tool. Participants were recruited through advertising and existing databases. Clinic visits occurred at baseline, 4 weeks, 8 weeks, 12 weeks, and 24 weeks. At all visits, the clinical trial manager recorded body weight, and participants then completed a computer-delivered survey, which measured psychological and lifestyle outcomes. Objective app usage data were recorded throughout the trial.

**Results:**

A total of 88 adults who had lost and maintained at least 5% of their body weight within the last 2 years were randomized (45 MotiMate and 43 control). Overall, 75% (66/88) were female, and 69% (61/88) completed week 24 with no differences in dropout by condition (χ^2^_1,87_=0.7, *P*=.49). Mixed models suggested no significant changes in weight or psychological outcomes over 24 weeks regardless of condition. Of 61 completers, 53% (32/61) remained within 2% of their starting weight. Significant increases occurred over 24 weeks for satisfaction with life and weight loss self-efficacy regardless of app condition. Diet and physical activity behaviors did not vary by app or week. Negative binomial models indicated that those receiving the full app remained active users of the app for 46 days longer than controls (*P*=.02). Users of the full version of the app also reported that they felt more supported than those with the control app (*P*=.01).

**Conclusions:**

Although some aspects of the intervention app such as usage and user feedback showed promise, there were few observable effects on behavioral and psychological outcomes. Future evaluation of the app should implement alternative research methods or target more specific populations to better understand the utility of the coping interface.

**Trial Registration:**

Australia New Zealand Clinical Trials Registry ACTRN12614000474651; https://www.anzctr.org.au/Trial/Registration/TrialReview.aspx?id=366120

## Introduction

### Weight Management

According to the World Health Organization, 1.9 billion adults were overweight or obese in 2016 [1]. In response to the challenge of weight management, many weight loss programs have been developed. Although many people have initial success in changing their dietary and/or physical activity behaviors to lose weight, few successfully maintain their lost weight over the longer term [2]. For example, only 20% of people from the National Weight Control Registry in the United States managed to maintain initial weight losses after 2 years [3]. Successfully maintaining weight loss for 2 to 5 years greatly increases the likelihood of longer term success [4], as does increasing the duration of exposure to the weight loss program [5]. However, it currently appears as though weight loss is regained in a linear fashion with few mitigating factors [6].

Given the significant challenge of weight loss maintenance, it is unsurprising that few previous interventions have sought to tackle this issue. Targeting self-regulation skills is 1 strategy that is commonly suggested to assist in weight loss maintenance [2,7]. Wing et al [8] report on a study targeting these very skills, which compared 3 groups: a control group, which received only a quarterly newsletter; a group that received face-to-face intervention; and a group that received a Web-based intervention. One of the core features for both intervention groups was a bathroom scale, which gave color-coded feedback, indicating whether participants had a weight gain of 1.4 kg or less (green), between 1.4 and 2.2 kg (yellow), or more than 2.2 kg (red). Those in the green zone were sent minor reinforcements (mainly through positive messages). Those in the yellow zone were instructed to use problem solving to get back on track, and those in the red zone were instructed to reinitiate weight loss attempts. The face-to-face group attended monthly meetings, whereas the Web-based group received social support and advice through a Web interface. Over 18 months, there was no difference in weight regain for the Web-based intervention (mean 4.7 kg [SD 8.6]) compared with the control (mean 4.9 kg [SD 6.5]). However, the proportion of participants who stayed within 2.3 kg of their starting weight (ie, within the green or yellow zones) was significantly higher in the Web-based intervention compared with the control (45.6% vs 27.6%).

### Weight Management Interventions Using Digital Technology

Recent technological progress has resulted in a shift from Web-based to mobile phone-based weight management interventions, with or without face-to-face support with some promising results [9,10]. Mobile phones could be used to extend the active duration of engagement with a weight management program, even through simple features such as a text message [11]. Therefore, apps may be a useful delivery mechanism for prolonging weight management attempts and, consequently, weight loss maintenance. Digital interventions are often described as more cost-effective and able to be wider reaching than more intensive face-to-face programs. As technology becomes more sophisticated, the ability to provide just-in-time intervention means that portable devices may also be able to provide intervention at critical times. Indeed, a review of just-in-time interventions suggested that portable devices may be useful to enhance cognitive behavioral therapy for weight loss programs [12]. Mobile phones also provide an avenue for regular self-monitoring, which have been strongly linked with successful behavior change, particularly in weight management [13,14].

### Combining Behavioral Strategies and Digital Technology for Weight Loss Maintenance

In addition to behaviors such as self-monitoring in weight loss maintenance, Elfhag and Rossner [15] recognize the importance of stress and coping. They define coping as, “cognitive and behavioral efforts used to manage external and internal demands...that exceed available resources.” They suggest that people who regain lost weight have poorer coping strategies, use more avoidant coping methods, and use eating to regulate their mood. More recently, this has also been observed in an Australian sample who maintained weight losses 4 months after a weight loss program. This group showed stronger problem-solving skills and described more planning events than those who gained weight over the same period [16]. Conservation of resources, self-regulation theory, and, more recently, ego depletion help to explain these observations [17,18]. These theories suggest that an individual has limited capacity to navigate stresses successfully through each day. When demand exceeds supply, individuals are likely to get off-track, particularly in relation to behaviors that are not yet habitual. The more coping and psychological resources a person possesses, the less likely it is that the demand-supply equation be disrupted, which puts less strain on a person. Considering multiple strategies are needed to overcome a single issue or hassle [19], it is not surprising that the more coping strategies or resources a person possesses, the more likely it is that they will find one that successfully helps them to sustain their desired behavior change (eg, eating better or exercising more).

Very few weight loss maintenance interventions exist, and none has incorporated simple weight loss maintenance strategies into a supportive program that also targets well-being. Positive well-being and optimism can improve resilience and the ability to problem solve [20,21]. It can also help restore resources after depletion [22] and is likely to be a critical factor in the maintenance of behavior. Therefore, the aim of this study was to develop and test a theoretically and evidence-based mobile phone intervention for weight loss maintenance. Previous authors have emphasized the importance of theory-based interventions that use scientific evidence and use the functionality of modern phones [23-25]. Specifically, we aimed to design and evaluate an app to improve psychological well-being, engagement with the intervention, and, ultimately, weight maintenance outcomes.

## Methods

### Overview

The study was approved by the Commonwealth Scientific and Industrial Research Organisation (CSIRO) Human Research Ethics Committee (14/02) in April 2014 and registered with the Australian New Zealand Clinical Trials Registry (ACTRN12614000474651). After being screened over the phone by the clinical trial manager, potential participants attended a study information session delivered by the principal investigator, received an information sheet, and then provided written consent to participate. A grocery voucher was given to participants at weeks 12 and 24 to thank them for their time (total 2×Aus $20 per participant). At the end of the study, participants could request access to the alternate app.

### Participants

Power calculations were based on changes in mood observed in our previous study [26]. In a sample with 44 females divided into 2 conditions, we were able to detect a moderate effect (0.45) for changes in mood. The initial aim was to recruit 150 volunteers to allow for 30% dropout [26,27] and the inclusion of males, which may increase the variability in observations. The primary method of recruitment was through an existing clinical research unit database owned by CSIRO, which included the contact details of people who had consented to be contacted about future research. This method was supplemented by local print advertising, promotional news stories, and unaddressed promotional pamphlets delivered by Australia Post. In final recruitment efforts, an external recruitment company was engaged.

Participants had to meet the following eligibility criteria: adults (aged 18 years or older), lost at least 5% of their body weight within the last 2 years, access to bathroom scales, want to continue or maintain their weight loss, own a mobile phone with an operating system appropriate for the app (iPhone or Android), and willing to attend a clinic in the central business district 5 times over 6 months. Like a previous study, we asked participants to verify previous weight loss with a signed statement by friend and health professional [8]. Exclusion criteria were pregnancy (or planning pregnancy), active cancer, and type I diabetes.

### Study Design

The trial was a 12-week, parallel, randomized, single-blind, controlled trial with 12-week follow-up. Participants were randomized to 1 of 2 groups (intervention or control) in a 1:1 ratio. The clinical trial manager allocated participants based on their ID using a random number generator. During randomization, subjects were balanced for sex, age, ownership of an iPhone (vs an Android), and obese versus not (based on self-reported information in the screening questionnaire). All participants received an app called MotiMate and were blinded regarding their allocation. None of the investigators were involved with participant allocation. Due to the collection of objective usage data (described further below), investigators could not be blinded surrounding participant allocation; some participants had app feature interactions only available on the MotiMate intervention app.

**Figure 1:**
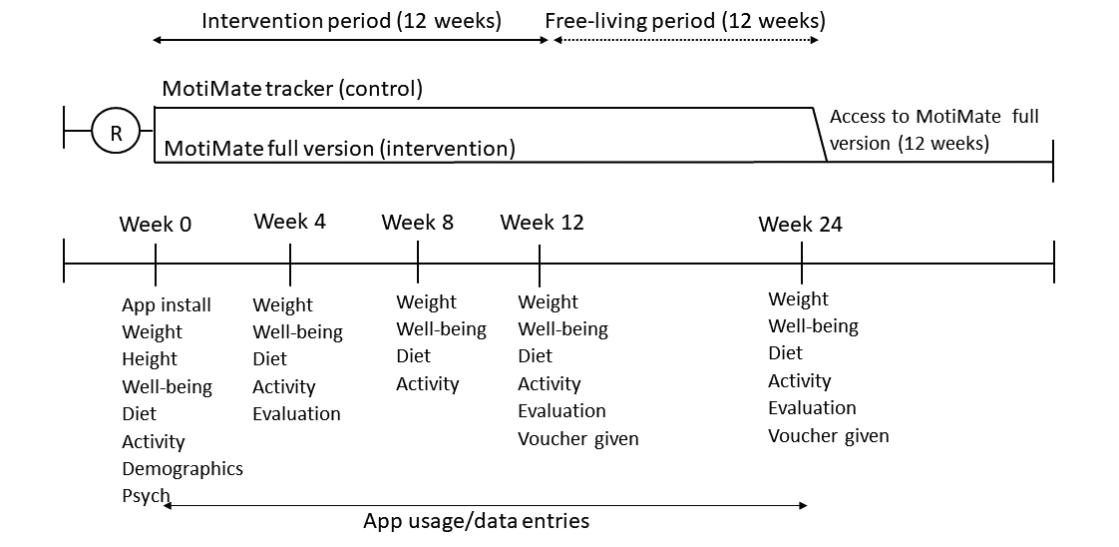
Study protocol. Activity: physical activity; Diet: diet quality; Evaluation: evaluation questions; Psych: self-esteem, restraint, satisfaction with life; Well-being: battery of measures.

Between late 2014 and mid-2015, participants made 5 visits to the clinical research unit in Adelaide, South Australia. Visits occurred at baseline (week 0), 4 weeks, 8 weeks, 12 weeks, and 24 weeks (Figure 1). At all visits, the clinical trial manager recorded body weight in kilograms, and participants then completed a computer-delivered survey that was programmed in SurveyMonkey (SVMK, Inc). These visits generally took less than 15 min each.

At the baseline visit, the intervention or control app was manually loaded onto the participants’ phones. Clinic staff confirmed correct allocation and recorded allocation in the participant clinic record. They then showed participants the app icon and ensured that participants could log in to the app using the account credentials entered at setup. To replicate a real-world setting where the app would be downloaded from an app store, clinic staff did not provide an overview of the app to participants.

### Interventions

Both groups received a mobile phone app designed by the research team to be used without any additional face-to-face support. The app was developed by an external company (Enabled) with close oversight from the study team and programmed for both iPhone and Android users. A helpline was established where technical enquiries or faults could be logged. The choice to make the intervention self-directed was partly to optimize cost-effectiveness and scalability but also because self-directed interventions have been shown to promote weight loss [28]. The content of the app did not change throughout the trial.

**Figure 2:**
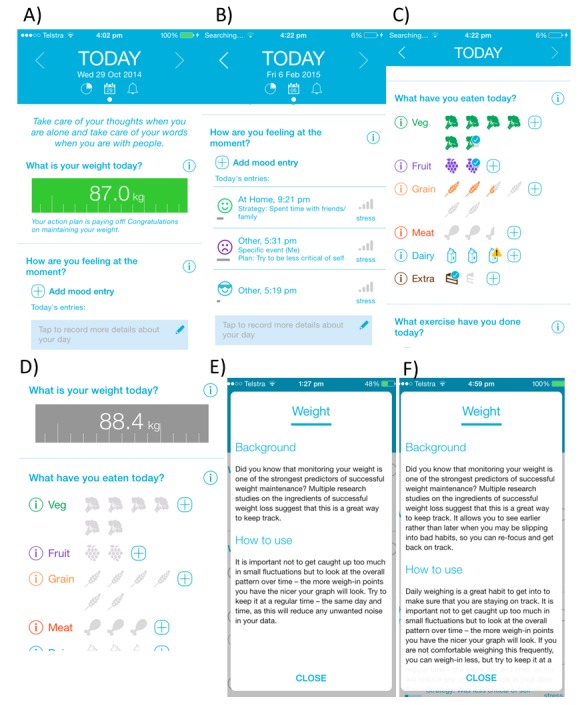
(A) Home screen for intervention app. (B) Mood entry interface for intervention app. (C) Food intake interface for intervention app. (D) Home screen for control app. (E) Information regarding weight tracking tool for control app. (F) Information regarding weight tracking tool for intervention group.

The development and features of the full version of the MotiMate app are described in detail elsewhere [29]. Briefly, both study apps had the same visual appearance, labeled MotiMate, and designed for daily use. The control version (also referred to as the tracker) included only features to track weight, food intake, and exercise. It primarily involved data entry with limited feedback and no encouraging/persuasive features (Table 1). The only feedback feature provided was a weight change graph. The full version of the app (or intervention version) was designed to include more persuasive and interactive features to help users track their weight, food intake, and physical activity and prompted users to enter data each day through notifications (Figure 2). In the intervention app, the food tracker gave users immediate feedback on whether they were meeting nutritional guidelines based on the number of serves of each food group that is recommended. Serving sizes were defined under the information tab. On the basis of the design used by Wing et al [8], the weight tracker in the intervention app also gave immediate feedback as to whether people were maintaining their weight (within 1.4 kg of their starting weight), gaining/in the danger zone (1.4 kg higher than starting weight), or had gained weight (2 kg or more over starting weight). These categories were indicated through colors, which went from green (maintaining) to faded green (gaining) to red (gained). The text displayed below the weight value also changed to be more directive with these categories. An automated email was sent to the study email address for participants who had entered a weight classified as gain. These people were contacted as soon as possible by a registered dietitian and asked if they had any questions or needed any advice. These phone calls were short and designed to provide just-in-time intervention to minimize further weight gain. Weekly summaries and graphs, which contextualized data entries in terms of success and areas for improvement, were also included in the intervention app.

Another major feature included in the full intervention app was a mood monitoring interface, which included a workshopping feature design to allow participants to develop their coping skills and emotional regulation. The workshopping interface was only triggered if a change in mood from positive to negative was detected or if a very positive mood became much less intense (large decrease in score). Once activated, users could workshop a cause of the change in their mood and then generate coping strategies to manage it. Once this process had been completed, if a positive change in mood was detected (from negative to positive), users then entered what had worked for them to help them change their mood. If users entered a highly negative mood (based on standard deviations) or prolonged negative mood states (of at least consecutive 7 days), an automated message was sent to the study email to contact this person regarding their mood. This was included to offer just-in-time intervention. In this instance, phone contact was made by a provisionally registered psychologist who followed a predefined protocol to assess if the user needed further support or referral to other services.

Despite creating slightly more cost and complication for a real-world translation of the MotiMate app, just-in-time contact with a registered dietitian and psychologist was included in the app to maximize its potential benefit. The ultimate vision for the app was it should be included as a tool as part of a wider health service with ready access to such professionals rather than employing these people specifically to support the app.

**Table 1:** Summary of core features of trial apps.

Feature	Intervention version	Control version
Daily notifications/prompts	The app sends a prompt to the user to remind them to enter data	Nil
System notifications	The system detects increases in weight or problematic mood patterns and emails the administrator who can then make person contact with the user to troubleshoot or direct to help	Nil
Weekly report	Summarizes all data entered each week and releases the report to the user	Nil
Motivational messages	At the top of the home screen, a motivational quote appears. The tone of these progresses with the duration of interaction	Nil
Weight entry	Participants slide the weight indicator to change their weight. Feedback is provided immediately with changes	Entry with no feedback.
Food entry	Users tap to indicate how much of food groups allowance they have consumed. A tick appears to indicate a satisfied group. An exclamation appears to indicate overconsumption	Entry with allowances but no tick or exclamation mark feedback
Diet action plans	System detects under- or overconsumption in certain target food groups and suggests that the user focuses on this area. In this interface, the user chooses from a prepopulated list of goals	Nil
Mood entry	Users can select from 6 different moods and then enter stress, location, and time	Nil
Mood change	The system detects negative changes or improvements in mood and triggers the coping workshop	Nil
Coping workshop	This guides people through planning how to overcome hassles (for negative change) or allows people to select coping strategies they did use to improve their mood (for improvement)	Nil
Exercise entry	Users enter duration, intensity, and type of exercise. They receive encouraging feedback for each entry	Entry with no feedback
Information text throughout	“i” buttons throughout the app give background information, instructions how to use features, and details about serving size for food groups	Same information with slightly less encouraging tone
Reviewing or entering data	The arrows at the top allow the user to navigate through previous data to enter data or review. Weight cannot be changed for previous days. Text at the top of the screen gives prompts encouraging more effective retrospective recall	Same as the intervention
Weight graph	Simple line graph showing changes in weight	Same as the intervention
Food graph	Summarizes food group intake according to whether each group is on target, over or under daily allowances	Nil
Exercise graph	Summarizes daily exercise entries according to moderate and intense minutes of exercise	Nil
Mood graph	Presents each mood recorded throughout the day. Moods can be tapped to see further details	Nil
Strategy graph	Summarizes all types of strategies used from the coping workshop into their parent groups: social, emotional, action, distraction, and others	Nil

### Technical Errors Throughout the Trial

Due to the technical issues with the app, an update was released after the first week of the trial. This affected iPhone users only. The update was released within 2 days of a reported fault. A total of 4 participants reported technical issues with their app. Two of these (did not see a weekly report and last data entered not saved) resolved themselves and may have been related to a temporary outage of the external database. The other 2 reports related to the app opening slowly and were for Android systems. The developers could not replicate this issue, and participants persisted with the app despite this inconvenience.

### Primary Study Outcomes

Unless stated otherwise, all outcomes were assessed at each visit (0, 4, 8, 12, and 24 weeks).

#### Subjective Well-Being

Subjective well-being was captured through 4 different variables including life satisfaction, depression, anxiety, stress, mood, and global happiness. Life satisfaction is considered an excellent indicator of a person’s total well-being and was assessed using the 5-item Satisfaction with Life Scale [30]. The short form of the Depression Anxiety Stress Scales was used to assess depression, anxiety, and stress [31]. Mood was captured using the validated and widely implemented 20-item Positive and Negative Affect Schedule [32]. Fordyce’s [33] simple 2-question scale was used to assess happiness. In addition to giving a total level of happiness between 0 and 10, respondents are asked to indicate the percentage of time that they feel happy, unhappy, and neutral.

#### Weight

The trial manager measured weight to 2 decimal places in the clinic using calibrated electronic scales (Mercury, AMZ 14) and standard operating procedures for collecting weight values.

### Other Outcomes

#### App Engagement/Evaluation

Interactions with the app including logging in and accessing each of the core features were captured by the app and sent to an external database. In a questionnaire, participants were also asked to complete a formal evaluation of each of the components of the app at weeks 4, 12, and 24. The evaluation assessed perceptions of features and opinions toward the app. Perceived usefulness, ease of use, and overall attitude to the app were assessed based on the Technology Acceptance Model [34], which is widely used to evaluate new technologies in the discipline of information systems. These data were largely descriptive in nature and are not reported in this paper.

#### Self-Efficacy

A total of 3 forms of self-efficacy (nutrition, exercise, and weight loss) associated with weight maintenance were measured. Nutrition and exercise self-efficacy were measured using the 10-item Nutrition and Physical Activity Self-Efficacy Scale [35]. These assess a person’s confidence in their ability to eat healthy foods and perform exercise in the presence of likely barriers. Weight loss self-efficacy refers to a person’s feeling that they can resist from eating in several different scenarios, such as when feeling emotional and distracted and in social settings. It was measured using the 20-item Weight Loss Self-Efficacy Scale [36].

#### Resilience

Resilience refers to a person’s belief in their ability to persist in the presence of difficulties. It is related to self-efficacy but encompasses a broader concept without being domain specific. To assess resilience, the 6-item scale, The Brief Resilience Scale, was used [37].

#### Coping

The 28-item Brief COPE questionnaire was used to assess coping style [38]. The tool assesses 14 different coping styles with higher scores representing greater use of each strategy. To minimize multiple analyses on each coping style, the subscales were factor analyzed (Multimedia Appendix 1). This indicated the presence of 2 factors used in the current analyses: the first included *Active* strategies (planning, active, reframing, emotional support, acceptance, and instrumental support), and the second included *Avoidant* strategies (denial, behavioral disengagement, substance use, and self-blame). Humor, religion, self-distraction, and venting did not load clearly on a single factor and were excluded. Factor scores were used to calculate an overall score for each of the 2 factors.

#### Lifestyle Behaviors

The 38-item short food survey gives a global score for diet quality out of 100 based on how well a person is meeting Australian Dietary Guidelines for the quantity, quality, and variety of different food groups [39]. The short form of the International Physical Activity Questionnaire was used to capture moderate, vigorous activity, and walking and sitting time throughout the previous 7 days [40]. It provides estimates for metabolic equivalent minutes, which represent a summary of total activity performed.

#### Demographics (Baseline Only)

Participants’ characteristics were captured using a standard medical questionnaire administered by the clinical research unit. Participants also completed several items describing their previous weight loss history.

### Confounding Variables (Measured at Baseline Only)

#### Self-Esteem

Self-esteem can influence many aspects of well-being [41]. It was assessed using the 10-item Rosenberg Self-Esteem Scale [42].

#### Dietary Restraint

To control for unwanted effects of dietary restraint, the 16-item Rigid Restraint Scale was used [43].

#### Neuroticism

Neuroticism describes the dispositional tendency to experience negative emotional states and is critical to outcomes such as mood. The 6-item Eysenck Personality Questionnaire Revised—Abbreviated was used to assess participants’ levels of neuroticism [44].

#### Dispositional Optimism

Dispositional optimism refers to a person’s tendency to have generally a more optimistic or positive outlook in the future. The 10-item Life Orientation Test was used to capture this [45]. Greater scores on this measure indicate higher levels of optimism relative to pessimism.

### Analyses

All analyses were performed in SPSS version 20 (IBM). The primary analyses involved intention-to-treat methods using mixed modeling to assess differences in well-being, weight, dietary intake, and physical activity levels over the study period between the intervention groups.

Given the smaller-than-desired final sample, preliminary bivariate correlations were used to assess the relevance of including all confounding variables. Dietary restraint was only weakly associated with a small number of the outcomes and, consequently, was not controlled for in any of the models. Neuroticism, self-esteem, and dispositional optimism (life orientation) related moderately to most of the psychological outcomes. For consistency, these variables were included in models assessing well-being, coping, resilience, and self-efficacy. All models also controlled for participants’ sex and age. The primary dependent variables were app condition, changes over time (by week), and the interaction between these 2 variables. In the presence of significant interaction effects between app condition and week, pairwise comparisons were made using Bonferroni adjustments.

Due to the skew in the app interaction data, comparisons of usage of features were made using negative binomial linear models. These models were overdispersed; therefore, the parameter model was estimated by SPSS rather than set to 1. App condition was compared controlling for sex and age in these models.

## Results

### Final Participants

**Figure 3:**
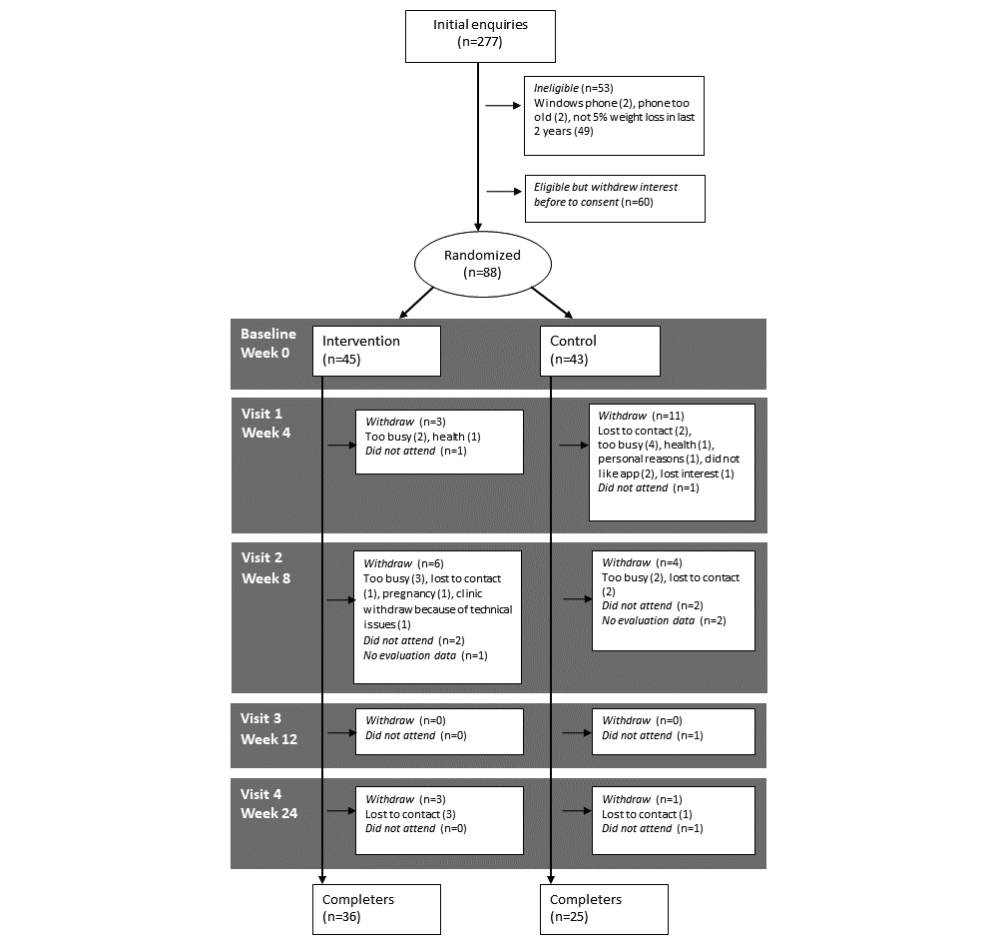
Consolidated Standards of Reporting Trial (CONSORT) participant flow diagram for trial.

Despite various recruitment attempts to reach 150 starters, 88 people started the trial (58.7% of 150 target), and 61/88 completed the trial (69% of starters). There were no differences in dropout by condition (χ^2^_1,87_=0.7, *P*=.49). Most people withdrew (n=11) because of being too busy with other commitments. Others were lost to contact (n=9; see Figure 3). On the basis of our previous observations [26], this number of completers should have provided 81.1% power to detect a moderate-sized difference in change in mood between groups and 97% power to detect a 2.5% difference in weight between groups.

**Table 2:** Participants’ demographics at trial commencement. No statistical differences were found between intervention and control groups for any categories.

Variables		Intervention (n=45)	Control (n=43)	Total
Sex (female), n (%)		33 (73)	33 (77)	66 (75)
Age, mean (SD)		44.5 (13.39)	45.8 (13.11)	45.13 (13.19)
**Weight status, n (%)**				
	Normal	10 (22)	7 (16)	17 (19)
	Overweight	20 (44)	15 (35)	35 (40)
	Obese class 1	8 (18)	13 (30)	21 (24)
	Obese class 2	1 (2)	4 (9)	5 (11)
	Obese class 3	6 (13)	4 (9)	10 (22)
iPhone ownership (vs Android), n (%)		31 (69)	31 (72)	62 (71)

The sample was between the ages 20 and 67 years and mostly female (66/88; 75%; Table 2). A majority owned an iPhone (62/88; 71%) rather than an Android handset. The group’s starting weight ranged from 53.4 to 170.4 kg with a mean of 85.8 kg (SD 22.08). Body mass index was between 20.9 and 60.8 kg/m^2^.

In terms of their weight maintenance, most of the sample (76/88; 86%) was currently below the heaviest weight they had been, but above the lowest weight they had ever been. At the start of the trial, participants reported being between 5.0% and 45.2% lighter than their maximum ever weight with 64% (56/88) maintaining at least a 10% loss from their maximum weight. The remaining 11 people in the sample were at or within 1% of their lowest weight when they started the trial.

### Primary Outcomes

#### Subjective Well-Being

**Figure 4:**
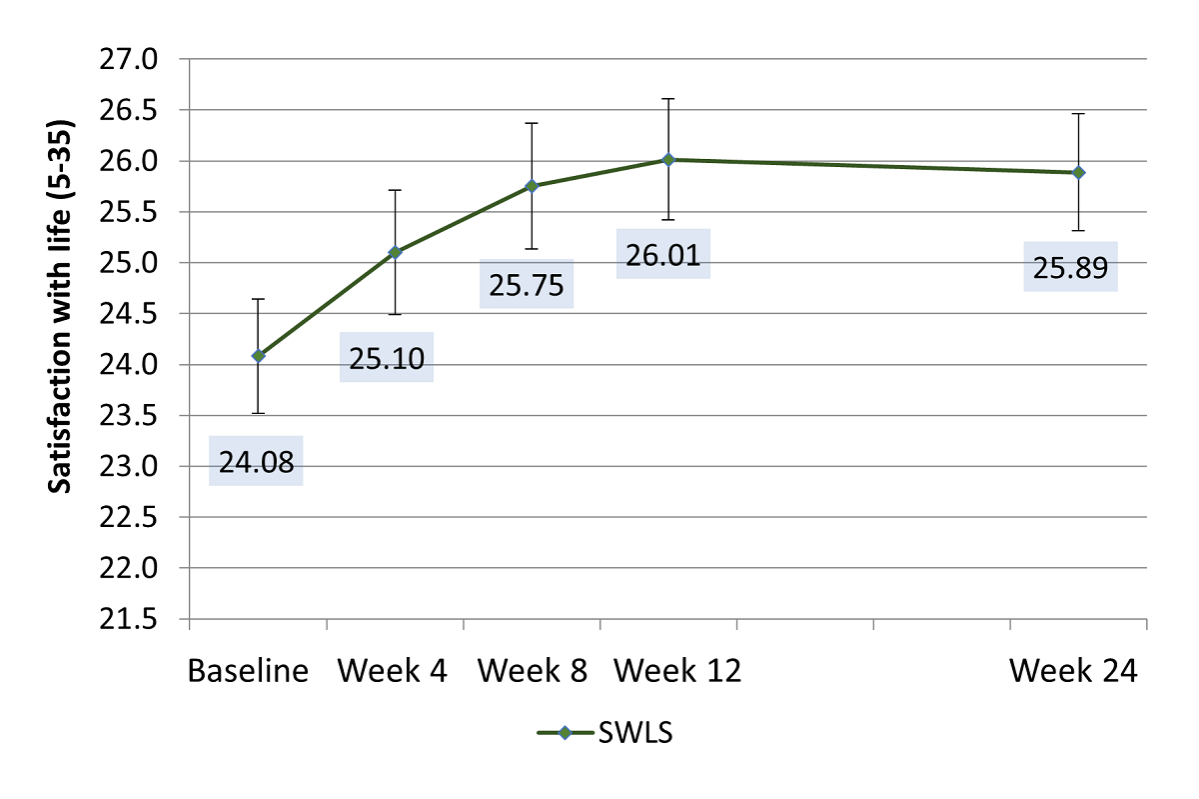
Adjusted means for Satisfaction With Life Score (SWLS) over the study period pooled for both intervention groups. Significant comparisons: baseline to week 8 (*P*=.046); baseline to week 12 (*P*<.01); and baseline to week 24 (*P*=.01). Means adjusted for participants’ sex and age, neuroticism, self-esteem, and dispositional optimism.

Satisfaction with life did not vary by app condition; however, there was a significant effect of time for the pooled means between groups (Figure 4). The differences between baseline and week 8 (*P*=.046), week 12 (*P*=.01), and week 24 (*P*=.01) were all significant. Means indicated that, for all study participants, life satisfaction improved over the 24-week study period.

Neither depression nor stress varied significantly by week or app condition (Multimedia Appendix 2). The interaction between app condition and week was significant for anxiety scores. Posthoc comparisons revealed no differences between the app conditions at any time point. The only significant pairwise comparison was for the large decrease in anxiety between baseline and week 4 for the control group (*P*=.02).

The interaction between app condition and time was also significant for negative affect. Scores for the control and intervention groups were significantly different at baseline (*P*=.02) and week 24 (*P*=.01), with the control group starting with significantly higher levels of negative affect and finishing the trial significantly lower. Although the control group had a significant reduction in negative affect between baseline and week 24 (*P*<.001) and week 12 and week 24 (*P*=.01), the intervention group had no differences in their negative affect levels throughout the trial (Multimedia Appendix 2). Positive affect, happiness, and the proportion of the time spent happy and unhappy did not vary by the app condition or week throughout the trial (Multimedia Appendix 2).

#### Weight

Most of the final sample (32/61; 53%) remained within ±2% of their starting weight at 24 weeks, with an average shift of less than 0.1% between baseline and week 24. At week 24, 41% (25/61) of participants who attended their final visit ended at the same weight or with a net loss (0% to −8.54%). The remaining 4 participants gained between 0.17% and 10.32% of their starting weight. There were no significant differences between the different app versions or over time for the percentage of weight change from baseline (Multimedia Appendix 2).

### Other Outcomes

#### App Usage

**Figure 5:**
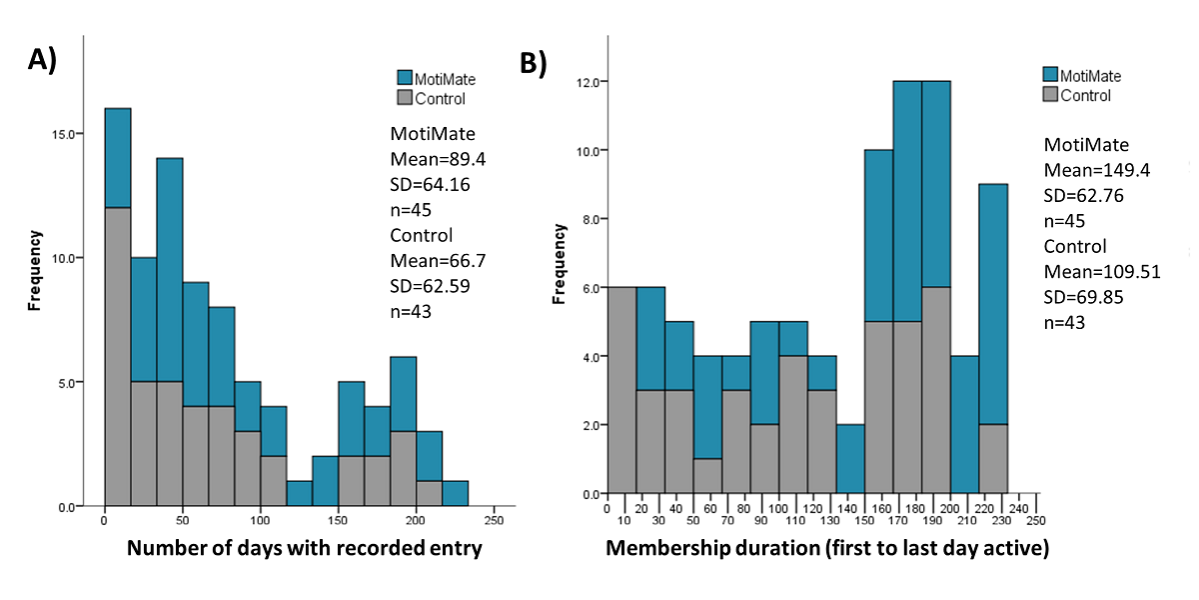
(A) Number of days data were recorded by the participants. (B) Participants’ overall membership duration (the amount of time between when the user started using the app and the final time they used it).

Those with the intervention app remained active users of the app for significantly longer than the control group, with a mean difference of almost 50 days (Table 3). Interactions with the app ranged between 0 and 168 days of the trial, with some users continuing their usage beyond their final visit (up to 223 days; Figure 5). The single user who had 0 days of membership was in the control group and received the app but never opened it before dropping out before their second visit.

**Table 3:** Adjusted means based on negative binomial models for food and exercise entries, number of days data were entered, and total days remaining active (membership days). Models adjusted for participants’ sex and age.

Variables	Intervention (n=45)		Control (n=43)		Wald chi-square	*P* value
	Mean	SE	Mean	SE		
Food entries	67.05	10.13	47.51	7.37	2.9	.09
Exercise entries	40.94	7.68	23.38	4.45	5.1	.02
Days data entered	87.04	11.86	62.69	8.95	3.2	.07
Membership days	151.24	16.91	105.42	12.43	5.7	.02

#### Data Entries

**Figure 6:**
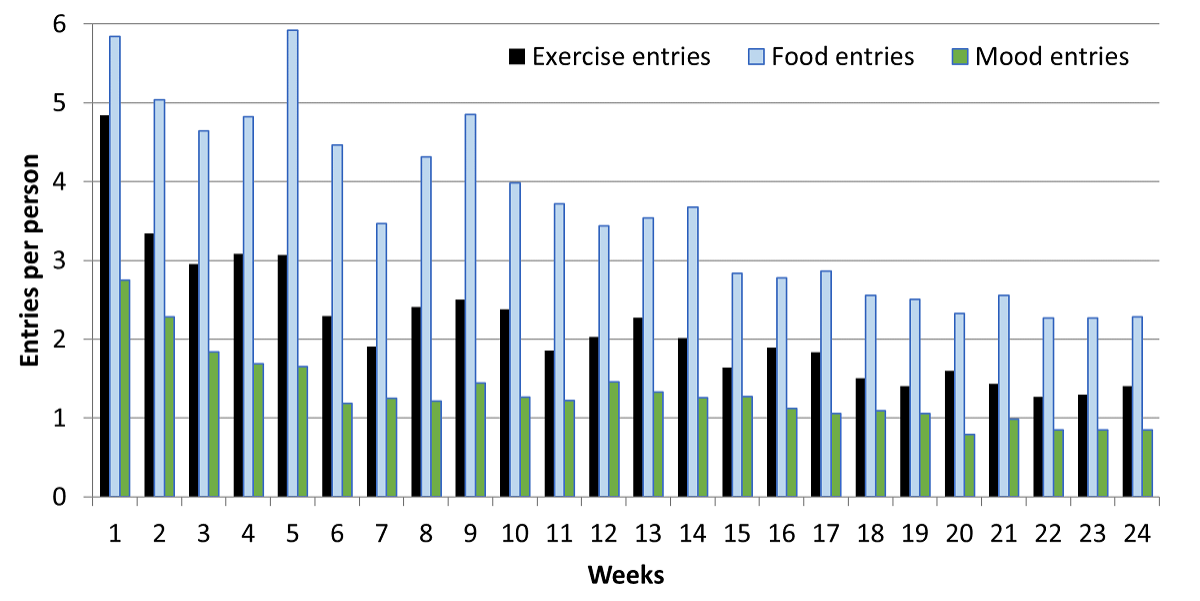
Number of data entries made per person each week over the study period for exercise, food, and mood (intervention group only).

In week 1, users created food entries on 5.8 out of a possible 7 days. By week 12, 3.3 entries, on average, were made per person, and during the free-living periods (weeks 12-24), between 2 and 3 entries per user were recorded per week (Figure 6).

The median food entry was 54 for the intervention group and 34 for the control. Both the intervention and control groups had 5 users contributing over 150 food entries. However, only 4 (8.9%) of the 45 intervention users made less than 10 food entries compared with 13 (30.2%) of the 43 control group users. Days that food entries were made only trended toward significance between the groups (Table 4).

Those receiving the intervention app made significantly more exercise entries relative to those receiving the control app (Table 3). The median exercise entry was 22 for intervention group and 12 for the control group. Exercise recording fell more sharply than food entry recording (Figure 5). Walking was by far the most popular exercise recorded (1836 entries) followed by weights/fitness classes (657 entries).

**Table 4:** Means for evaluation ratings of the app presented by the intervention and control app groups.

Variables		Intervention		Control		*P* value
		n	Mean (SD)	n	Mean (SD)	
**Week 4**						
	The app helped me control my weight	39	3.41 (0.906)	31	3.16 (1.003)	.28
	I have enjoyed using the app	39	3.67 (0.898)	31	3.42 (1.148)	.32
	Ease of use of app (TAM^a^)	39	3.69 (0.919)	31	3.94 (0.859)	.25
	Perceived usefulness of app (TAM)	39	3.69 (0.919)	31	3.94 (0.859)	.23
	Attitude to app (TAM)^b^	39	3.96 (0.818)	31	3.53 (0.890)	.04
**Week 12**						
	The app helped me to be more aware of my eating	34	5.44 (1.501)	29	5.03 (1.592)	.30
	The app helped me to be more aware of my exercise	34	5.24 (1.671)	29	4.55 (1.703)	.11
	The app helped me to be more aware of my weight^b^	34	5.62 (1.415)	29	4.72 (1.709)	.03
	The app has supported me^b^	34	5.38 (1.518)	29	4.17 (1.872)	.01
**Week 24**						
	What score out of 10 would you give the app?^b^	32	6.38 (2.012)	28	5.04 (2.333)	.02

^a^TAM: Technology Acceptance Model.

^b^Significant differences.

In the intervention group, there was a wide range of interactions with the mood monitoring feature with a median of 20 mood entries per person over the 24 weeks. In total, 2346 mood entries were made. More than 50% of the intervention sample (23/45) had less than 20 mood entries throughout the study period. There was a small, but very active, group of users (7/45, 16%) who made more than 100 mood entries. Overall, mood recording was used less than the traditional food and exercise monitoring tools with 2.75 entries per person in week 1 and a steep decline in entries even over the first few weeks (Figure 5). The most commonly entered mood was happy (924/2346, 39.38%), followed by relaxed (n=500/2346, 21.31%) and positive (497/2346, 21.18%).

### Use of Persuasive Features (Intervention Group Only)

Only 2 users were contacted by a provisionally, registered psychologist, who was part of the wider study team, because of entering a pattern of highly negative moods—one reported having a relationship break up, and the other reported suffering from posttraumatic stress disorder.

Furthermore, 5 users were contacted by a dietitian regarding weight gains. These were largely around the Christmas holiday period, and participants generally did not want specific help or advice, generally saying, “they knew what they needed to do.”

In addition, 25 (56%) of the 45 participants in the intervention group received a diet action plan (147 action plans generated). Only 43 of the 147 plans (29.3%) were marked as completed by users. The most common plans triggered were those relating to underconsumption (87/147). This may be because people had not entered food data for these days. This was followed by messages regarding excessive discretionary foods (n=25) and not enough fruits and vegetables consumption (n=18).

Only 3.87% (91/2346) of all moods entered had an associated workshop entry recorded. Hassles could be entered if users selected “Tell us more” to enter the coping workshop. In 2254 cases, users answered “Dismiss” to this question. A total of 92 coping workshop entries were made. The most frequent hassle was “Nothing in particular” (22%, 20/92), followed by “People problems” (20%, 18/92).

#### Self-Efficacy, Resilience, Coping, Diet, and Activity

**Figure 7:**
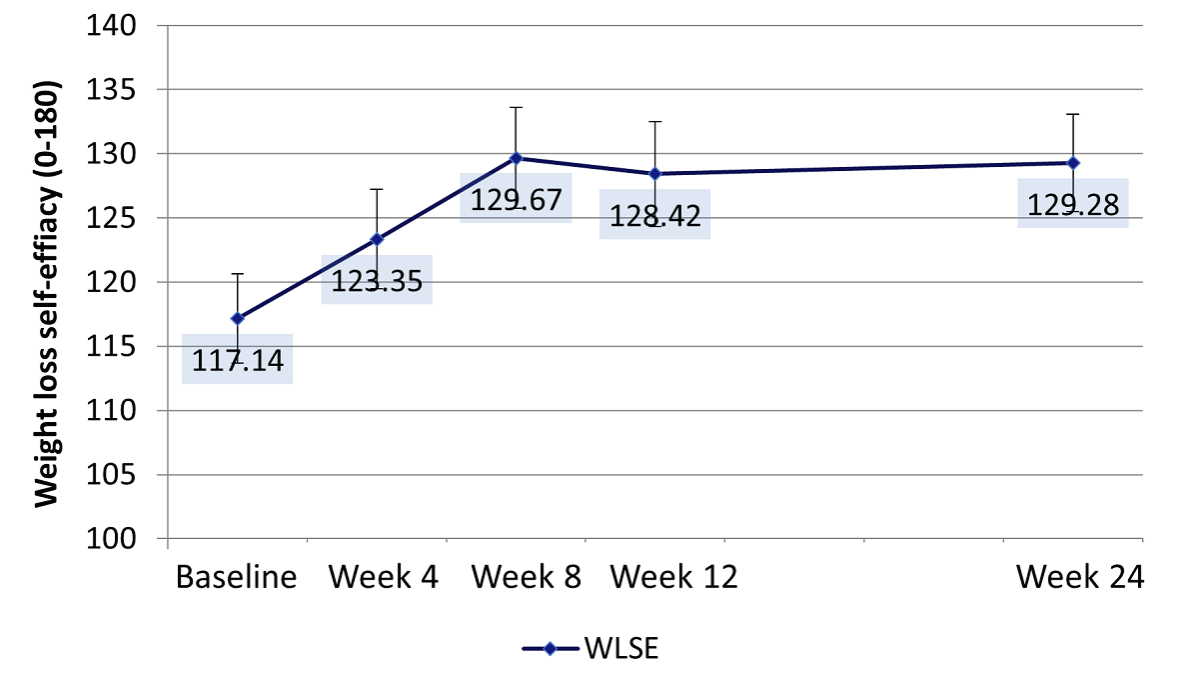
Adjusted means for weight loss self-efficacy (WLSE) over the study period. Significant comparisons baseline and week 8 (*P*=.001), week 12 (*P*<.01), and week 24 (*P*<.001).

Weight loss self-efficacy only differed significantly by week (Multimedia Appendix 3). Increases between baseline and week 8 (*P*=.001), week 12 (*P*<.01), and week 24 (*P*<.001) were significant (Figure 7).

There was a significant interaction effect between app condition and week for resilience (Multimedia Appendix 3). The strongest difference between apps at any time point was at week 12, where the intervention group had higher resilience than the control group. However, this failed to reach significance (*P*=.08). The interaction effect appeared to be driven by significant differences between weeks within app condition. The control group had an initial improvement in resilience with a significant difference between values at baseline and week 8 (*P*=.04). The intervention group had a significant decrease in resilience in the free-living period (from weeks 12 to 24; *P*=.02). These were the only significant pairwise comparisons.

There were no differences for coping styles, diet quality, and physical activity between app condition, time, or the interaction between the 2 (Multimedia Appendix 3).

### App Feedback

**Figure 8:**
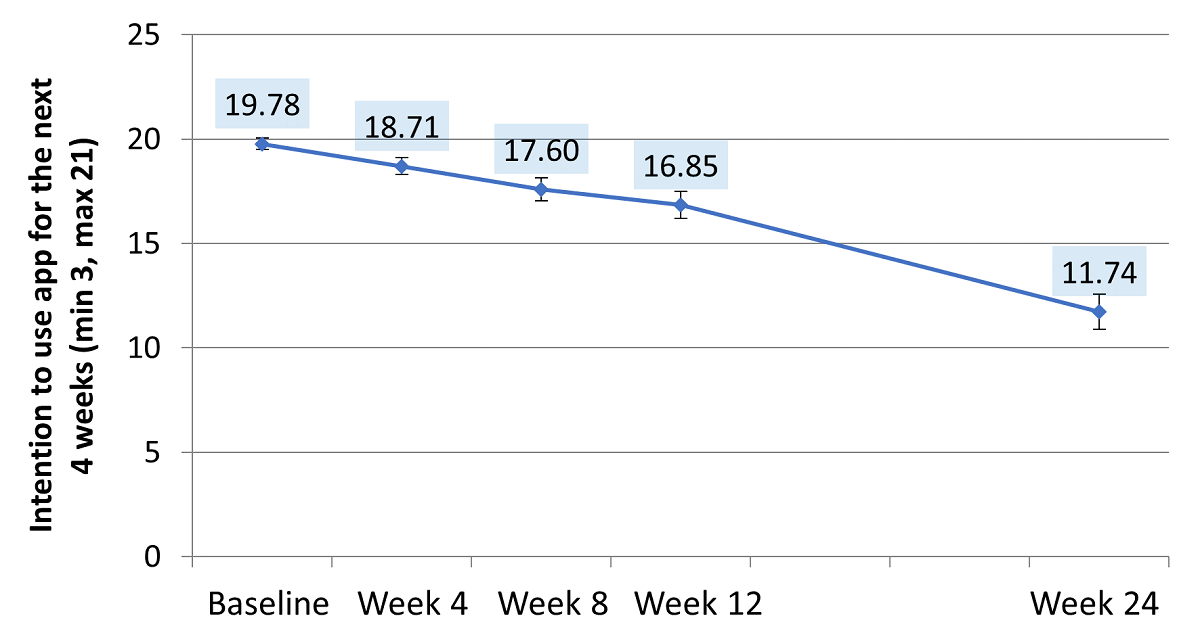
Intention to use app for the next 4 weeks. Adjusted means based on mixed models, pooled across app condition. Changes from baseline to week 8 onward were all significant (all *P* ≤.005). min: minimum; max: maximum.

At week 4, most of the intervention app users would have recommended the app to a friend (32/39, 82% *yes*; 4/39, 10% *no*; and 3/39, 8% *other*). In contrast, less than half of the participants agreed that they would recommend the control app (14/31, 45 *yes*; n=12/31, 39% *no*; 5/31, 16 *other*). This difference was significant (χ^2^_2_=10.7, *P*<.01). A mixed models comparison of the average scores across the study showed that the intention to continue using the app had fallen significantly by the end of 24 weeks (*F*_4, 66.9_=22.74, *P*<.001; Figure 8). Pairwise comparisons revealed that changes from baseline to week 8 onward were all significant (all *P*<.01). Changes did not differ by the app conditions (Figure 8).

There were differences between the intervention groups for attitudes toward the app, how much users felt the app supported them, and how the app assisted users in being aware of their weight (Table 4). At the end of the study, the overall rating given to the intervention app was also significantly higher.

## Discussion

### Summary of Results

The MotiMate app was designed to provide support for those undertaking weight loss maintenance. It was designed to be cost-effective with minimal personal contact and support people in tracking their weight, food intake, exercise, and moods. In our highly controlled, 6-month clinical trial of the MotiMate app, we were unable to show any additional benefits of persuasive features and mood monitoring in terms of psychological well-being and weight maintenance for participants. This is despite observations of longer engagement with the app, more exercise entries, and more positive rating of the intervention app by users. There were improvements in weight loss self-efficacy and life satisfaction throughout the trial in both groups. These are important constructs for well-being and weight maintenance. Interaction effects were observed for anxiety and negative affect. However, posthoc analyses revealed that these may have been driven by baseline differences and not the intervention *per se*. A significant interaction effect for changes in resilience was also observed with the intervention group having significant falls in the free-living period, whereas the control group did not. There were minimal differences observed in lifestyle behaviors and other subjective well-being constructs.

### Weight Management

Most participants maintained their weight regardless of app condition over 24 weeks, with more than half remaining within 2% of their starting weight. On face value, this seems like a positive outcome, especially for weight loss maintenance, which is notoriously challenging. This result supports a previous study by Wing et al, which indicated that 53% of their control group maintained weight at 6 months [8]. Their control group was much less active than the one used in this study, as their participants only received newsletters, whereas our control participants received a monitoring app.

At the start of our trail, participants were within different ranges from their lowest ever weights, and they each had different time frames with which they had been maintaining their weight, as well as different experiences with weight loss programs before starting the study. It would have been interesting to explore how these factors may have altered weight outcomes; however, our ability to do this was limited because of the sample size. The choice to recruit people with a range of weight management experiences was a purposeful one to assess if the MotiMate design could be effective in a real-world setting, where people have had a variety of weight loss experiences; however, this may have also added increased variability to the outcomes. Close to 40% of people continued to lose weight while on this trial, although they had no support specifically directed toward weight loss from the app. There were no differences over time or between apps for diet quality and exercise, which suggest that these people also did not significantly change their lifestyle practices. Therefore, some of these people may have been still engaging in active weight loss efforts to try to overcome a plateau in weight loss rather than maintain an existing weight. Unfortunately, we did not explicitly capture this intention at study commencement. However, recruitment materials and study information all focused on maintaining weight loss rather than losing weight.

Capturing people in the small window between weight loss and regain was more difficult than anticipated. This may be avoided by first placing people into a weight loss program in the future. Although we did not hit our recruitment target, the final sample size still provided adequate power to detect moderate effects.

### Mood Features and Psychological Changes

Engagement with the app features related to mood was low. This is likely to explain the absence of differences between the 2 groups. Even more so, given that the control group also received an app with monitoring features. Therefore, these participants received a more active intervention than a standard usual care model in which they may only have been given once-off advice or static information such as newsletters and pamphlets. The low engagement with mood features may be partly because of the study design and the desire to blind participants as to their allocation. No mention was made regarding mood monitoring in recruitment. Qualitative feedback (not reported) indicated that some people were not receptive to tracking their mood. Moreover, 1 participant even indicated that they only ever had *one mood*, and there was *no need to record it*. Indeed, a review of emotion research suggested individual variability in emotional granularity [46]. Trialing the app in an uncontrolled sample would allow us to target a potentially more appropriate market in the future.

It is unclear why those in the intervention group had a significant fall in resilience in the free-living period. Although, it is important to note that this change was observed within this group, and the difference between resilience scores was not significant between the apps. We observed improvements in measures of well-being throughout the trial that have not been documented in many previous studies. Yet, recent studies reinforce the idea that well-being is a critical factor for weight loss maintenance [47], and apps using behavior change techniques relating to problem solving and stress reduction are needed [48]. However, it may also be that weight change alone may be crucial for changes in coping and problem-focused coping [49]. Therefore, the ability of simple behavioral therapy techniques may not be able to add value to weight loss alone. That is not to underplay the potential importance of behavioral therapies for improving adherence to lifestyle programs [50] and the potential benefits of improving behavioral skills before engaging in a weight management program [51].

Since this study started, recent evidence has emerged that suggests that resource depletion theory may not be as strong as has been previously thought [52]. More recent studies have failed to replicate observations consistent with ego depletion [53,54] and have called in to question the presence of the described effects. Ego depletion is a relatively new theory, and further studies may be needed to better understand ego depletion and its relationship to eating habits and weight management. Emotion regulation strategies may benefit those prone to emotional eating more observably than other groups.

### MotiMate App Ratings and Engagement

The participants had significantly more positive attitudes to the intervention app relative to the control with 82.1% agreeing that they would be happy to recommend the full version of MotiMate to a friend. Intervention users also felt that the app helped them to be more aware of their weight and felt more supported than people using the control app. Nonetheless, motivation to engage with the app fell for both groups by the end of the trial. However, taken with usage data, those with the intervention app continued engaging with the app longer than those with the control app. Engagement with the intervention app features was also higher relative to the control group. Despite previous papers suggesting that app use is associated with better weight loss results [55] and that extended contact through mobile phone improves weight loss maintenance [56], better app use did not translate to better weight loss maintenance in this instance. This once again may be related to how long participants had been successfully maintaining their weight before the trial.

Engagement with the app and intention to continue using fell over 6 months for both apps. Aside from early drops in usage, there was a visible decrease in motivation at week 8. This is an observation that we have made in similar trials [26]. To improve the testing of app-based programs in the future, alternative methods of evaluation may be needed including adaptive intervention designs [57]. It is also important to note that although participants may not be recording their behaviors into the app, this does not necessarily mean that they have not performed these behaviors. It is likely that there is a point where behaviors such as diet monitoring become habitual, and there is no need to rely on tools for assistance. In a real-world translation of MotiMate, usage could be tracked, and just-in-time contact could be made with users when their interactions fall in an effort to understand why they have stopped using the app or to prompt them to keep using the app. This may help to mitigate disengagement because of perceived failure (eg, not entering weights because there has been a gain).

### Conclusions

Although some aspects of the MotiMate app showed promise, there were few observable effects of using the full intervention app relative to the basic tracker. Future evaluation of the app may need to be implemented using more progressive research methods or targeting a larger or more specific population to better understand the utility of the coping interface.

## Appendix

Multimedia Appendix 1 Factor analysis of COPE inventory.

Multimedia Appendix 2 Adjusted means and standard errors (SE) for primary outcomes presented by week and app condition.

Multimedia Appendix 3 Adjusted means and standard errors (SE) for secondary study outcomes.

Multimedia Appendix 4 CONSORT-EHEALTH checklist (V 1.6.2).

## Abbreviations

BMI: body mass index
CSIRO: Commonwealth Scientific and Industrial Research Organisation
MET: metabolic equivalent
SD: standard deviation
SWLS: Satisfaction With Life Scale
TAM: Technology Acceptance Model
WLSE: Weight Loss Self-Efficacy
