# Supplementary material for: A Mobile Phone App Designed to Support Weight Loss Maintenance and Well-Being (MotiMate): Randomized Controlled Trial
Source: JMIR Mhealth Uhealth. 2019 Sep 4;7(9):e12882. doi: 10.2196/12882 (PMC6834303; doi:10.2196/12882)
Supplement: Multimedia Appendix 1 [file mhealth_v7i9e12882_app1.pdf]

Multimedia Appendix 1. Factor analysis of COPE inventory.

As recommended by the author of the COPE (Carver, Scheier, & Weintraub, 1989), sub-scales were subjected to factor analysis to reduce the number of coping outcomes to be analysed. Using Principle Components Analysis (PCA), initial extraction suggested 4 higher order factors according to Eigenvalue criteria. However, Monte Carlo Parallel Analysis suggested that only 2 factors were optimal. Due to cross-loadings on both factors, coping styles of humor, religion, self-distraction and venting were removed from the analysis. Due to low correlation between the extracted factors, Varimax rotation was used to generate the final factor loadings (presented right).

These factor loadings were used to generate factor scores for each participant on each factor. Factor 1 included many 'active' coping strategies with planning, cognitive reframing and general active coping strategies being the top 3 loading items. Factor 2 included strategies associated with avoidance with denial, behavioural disengagement and substance use loading most strongly on this factor.

Table: Final results for factor analysis of the higher order factors from the COPE

	Factor 1 'Active'	Factor 2 'Avoidant'
**COPE planning score**	.803	
**COPE active coping score**	.788	
**COPE reframing score**	.781	
**COPE use of emotional support score**	.654	.246
**COPE acceptance score**	.645	
**COPE use of instrumental support score**	.611	
**COPE denial score**		.819
**COPE behavioural disengagement score**	-.135	.800
**COPE substance use score**		.628
**COPE self-blame score**	.196	.490

Extraction Method: Principal Component Analysis.
Rotation Method: Varimax with Kaiser Normalization.
Values lower than .10 suppressed

References

Carver, C. S., Scheier, M. F., & Weintraub, J. K. (1989). Assessing Coping Strategies - a Theoretically Based Approach. *J Pers Soc Psychol, 56*(2), 267-283. doi:Doi 10.1037//0022-3514.56.2.267
