# Supplementary material for: A Mobile Phone App Designed to Support Weight Loss Maintenance and Well-Being (MotiMate): Randomized Controlled Trial
Source: JMIR Mhealth Uhealth. 2019 Sep 4;7(9):e12882. doi: 10.2196/12882 (PMC6834303; doi:10.2196/12882)
Supplement: Multimedia Appendix 2 [file mhealth_v7i9e12882_app2.pdf]

Multimedia Appendix 2. Adjusted means and standard errors (SE) for primary outcomes presented by week and app condition.

Variables	Intervention					Control					Model outcomes		
Week	0	4	8	12	24	0	4	8	12	24	Time	Intervention	Interaction
Satisfaction with Life (SWLS)[a]													
Mean	25.41	25.75	26.7	26.47	26.01	22.75	24.45	24.8	25.56	25.76	F(4,63.0)= 3.96, P<0.01	F(1,75.2) =2.58, P=0.11	F(4,63)=2.09, P=0.09
SE	0.75	0.8	0.81	0.78	0.76	0.77	0.86	0.87	0.83	0.8			
Depression[a]													
Mean	1.82	1.85	2.77	2.22	2.62	2.36	2.18	1.92	2.77	2.36	F(4,63.0)= 0.82, P=0.52	F(1,59.4) =0.03, P=0.86	F(4,63.0) =1.99, P=0.11
SE	0.33	0.32	0.37	0.48	0.54	0.34	0.35	0.41	0.51	0.57			
Anxiety[a]													
Mean	2.15	1.97	1.85	1.66	1.78	2.6	1.43	2.17	2.24	1.81	F(4,63.9)= 2.42, P=0.06	F(1,70.4) =0.20, P=0.66	F(4,63.9) =2.69, P=0.04
SE	0.33	0.33	0.35	0.38	0.39	0.34	0.36	0.37	0.41	0.42			
Stress[a]													
Mean	4.99	4.81	4.23	4.7	4.89	4.45	4.05	4.23	5.02	3.78	F(4,65.5)= 1.16, P=0.34	F(1,69.7) =0.52, P=0.47	F(4,65.5) =0.75, P=0.56
SE	0.53	0.48	0.56	0.66	0.58	0.54	0.52	0.61	0.71	0.62			
Positive Affect[a]													
Mean	36.67	36.5	35.48	36.39	35.39	35.69	35.29	35.91	34.36	34.86	F(4,65.3)= 0.69, P=0.41	F(1,75.5) =0.51, P=0.73	F(4,65.4) =0.42, P=0.79
SE	0.92	0.91	1.21	1.22	1.1	0.94	0.99	1.32	1.31	1.15			
Negative Affect[a]													
Mean	16.76	16.9	17	16.98	17.99	19.11	17.26	17.3	18.55	14.8	F(4,65.4)= 1.90, P=0.12	F(1,72.0) =0.12, P=0.73	F(4,65.5) =5.14, P=0.01
SE	0.69	0.65	0.83	0.94	0.87	0.71	0.71	0.9	1.01	0.93			
Happiness score[a]													
Mean	3.17	3.1	3.08	3.08	3.28	3.48	3.21	3.05	3.28	3.38	F(4,69.2)= 2.36, P=0.06	F(1,74.7) =0.60, P=0.44	F(4,69.3) =0.72, P=0.58
SE	0.18	0.14	0.13	0.16	0.2	0.19	0.15	0.14	0.17	0.21			
Percentage time happy[a]													
Mean	65.48	68.96	68.59	68.94	66.6	65.33	62.54	62.55	64.17	63.99	F(4,63.5)= 0.24, P=0.91	F(1,76.9) =1.30, P=0.26	F(4,63.5) =1.19, P=0.32
SE	2.91	2.82	3.04	2.89	3.49	2.99	3.03	3.29	3.07	3.72			
Percentage time unhappy[a]													
Mean	14.52	13.92	12.48	13.06	12.68	14.19	15.73	13.55	13.61	12.11	F(4,65.5)= 1.37, P=0.25	F(1,73.3) =0.14, P=0.71	F(4,65.6) =0.44, P=0.78
SE	1.34	1.29	1.27	1.44	1.4	1.38	1.42	1.41	1.57	1.53			
Percentage weight change[b]													
Mean	na	-0.64	-0.55	-0.09	0.39	na	-0.57	-0.34	-0.40	-0.52	F(3,61.8)= 0.84, P=0.48	F(1,69.2) =0.22, P=0.64	F(3,61.8) =0.78, P=0.51
SE	na	0.25	0.38	0.44	0.57	na	0.27	0.43	0.48	0.62			

Significant comparisons; SWLS: Baseline to Week 8 (P=.046); Baseline to Week 12 (P<.01); Baseline to Week 24 (P=.01); Anxiety: Baseline to Week 4 (P=.02) in the control group; Negative Affect: Intervention and control different at Baseline (P=.02) and Week 24 (P=.01) Baseline to Week 44 (P<.001) and week 12 to week 24 (P=.01) in the control group.

[a]Means adjusted for participant sex and age, neuroticism, self-esteem and dispositional optimism.

[b]Means adjusted for sex and age only.
