# Supplementary material for: A Mobile Phone App Designed to Support Weight Loss Maintenance and Well-Being (MotiMate): Randomized Controlled Trial
Source: JMIR Mhealth Uhealth. 2019 Sep 4;7(9):e12882. doi: 10.2196/12882 (PMC6834303; doi:10.2196/12882)
Supplement: Multimedia Appendix 3 [file mhealth_v7i9e12882_app3.pdf]

Multimedia Appendix 3. Adjusted means and standard errors (SE) for secondary study outcomes.

Variables	Intervention					Control					Model outcomes		
Week	0	4	8	12	24	0	4	8	12	24	Time	Intervention	Interaction
Weight Loss Self-Efficacy (WLSE)[a]													
Mean	120.39	125.57	131.26	130.26	126.94	113.90	121.12	128.08	126.59	131.61	F(4,63.5) =5.99, P<.001	F(1,81.1) =0.18, P=.67	F(4,63.5) =1.47, P=.22
SE	4.58	5.10	5.13	5.38	5.01	4.71	5.39	5.48	5.70	5.25			
Nutrition Self-Efficacy[a]													
Mean	15.84	16.27	16.35	16.7	16.37	15.56	15.94	16.06	16.37	15.7	F(4,67.9)= 1.48, P=0.217	F(1,79.7) =0.56, P=0.458	F(4,67.9) =0.12, P=.97
SE	0.43	0.46	0.49	0.47	0.48	0.44	0.5	0.53	0.51	0.51			
Exercise Self-Efficacy[a]													
Mean	16.00	15.70	15.51	16.07	16.03	14.51	14.23	14.56	15.08	14.62	F(4,66.0)= 0.71, P=.59	F(1,77.3) =3.65, P=.06	F(4,66.0) =0.24, P=.92
SE	0.49	0.56	0.64	0.69	0.6	0.51	0.6	0.69	0.74	0.63			
Resilience[a]													
Mean	3.74	3.79	3.86	3.96	3.67	3.63	3.78	3.87	3.74	3.89	F(4,63.3)= 3.62, P=.01	F(1,76.7) =0.05, P=.82	F(4,63.3) =2.87, P=.03
SE	0.08	0.08	0.09	0.09	0.1	0.08	0.09	0.1	0.09	0.11			
Coping Factor 1 (Active)[a]													
Mean	0.07	0.15	0.15	0.11	-0.01	0.01	-0.15	-0.19	-0.08	0.01	F(4,60.8)= 0.07, P=.99	F(1,67.1) =0.89, P=.35	F(4,60.8) =0.86, P=.49
SE	0.16	0.17	0.18	0.2	0.18	0.16	0.17	0.19	0.21	0.19			
Coping Factor 2 (Avoidant)[a]													
Mean	0.09	0.11	0.15	0.09	0.21	-0.01	-0.11	-0.01	0.04	-0.10	F(4,64.0)= 0.09, P=.99	F(1,66.0) =1.33, P=.25	F(4,64.0) =0.31, P=.87
SE	0.12	0.15	0.17	0.18	0.16	0.12	0.16	0.18	0.18	0.17			
Diet Quality[b]													
Mean	55.4	56.54	58.73	59.45	56.96	55.67	55.85	55.41	55.94	57.11	F(4,63.8)= 1.13, P=.35	F(1,80.5) =0.47, P=.50	F(4,63.8) =1.20, P=.32
SE	1.59	1.68	1.92	1.76	1.84	1.65	1.82	2.07	1.88	1.94			
Total MET (mins exercise)[b]													
Mean	9236.21	9561.61	10403.21	9344.5	9373.06	8295.24	8230.25	8092.32	7167.17	9664.6	F(4,66.2)= 1.05, P=.39	F(1,80.2) =1.78, P=.19	F(4,66.2) =1.17, P=.33
SE	834.45	781.33	1103.39	925.08	905.77	868.02	846.47	1213.03	986.33	953.1			

[a]Means adjusted for participant sex and age, neuroticism, self-esteem and dispositional optimism.

[b]Means adjusted for sex and age only
