# Supplementary material for: A Mobile Phone App Designed to Support Weight Loss Maintenance and Well-Being (MotiMate): Randomized Controlled Trial
Source: JMIR Mhealth Uhealth. 2019 Sep 4;7(9):e12882. doi: 10.2196/12882 (PMC6834303; doi:10.2196/12882)
Supplement: Multimedia Appendix 4 [file mhealth_v7i9e12882_fig.pdf]

CONSORT-EHEALTH Checklist V1.6.2 Report	Manuscript Number	12882
**(based on CONSORT-EHEALTH V1.6), available at [http://tinyurl.com/consort-ehealth-v1-6].**		
**Date completed**		
3/12/2019 0:29:28		
**by**		
Emily Brindal		
A smartphone phone application designed to support weight loss maintenance and wellbeing: Results of a randomized trial for the MotiMate app		
TITLE		
**1a-i) Identify the mode of delivery in the title**		
A smartphone phone application		
**1a-ii) Non-web-based components or important co-interventions in title**		
not relevant, delivered through app only		
**1a-iii) Primary condition or target group in the title**		
weight loss maintenance		
ABSTRACT		
**1b-i) Key features/functionalities/components of the intervention and comparator in the METHODS section of the ABSTRACT**		
"The intervention app included more persuasive and interactive features to help users track their weight, food intake and physical activity and prompted users to enter data each day through notifications and included a mood and stress workshopping tool."		
**1b-ii) Level of human involvement in the METHODS section of the ABSTRACT**		
"Both study apps had the same visual appearance and were designed to deliver all intervention content without face-to-face contact."		
**1b-iii) Open vs. closed, web-based (self-assessment) vs. face-to-face assessments in the METHODS section of the ABSTRACT**		
"Participants were recruited through advertising and existing databases.<br>At all visits the clinical trial manager recorded body weight and participants then completed a computer-delivered survey which measured psychological and lifestyle outcomes. "		
**1b-iv) RESULTS section in abstract must contain use data**		
"Eighty-eight adults who had lost and maintained at least 5% of their body weight within last 2 years were randomised (45 MotiMate, 43, control). Of 88 starters, 75% (n=66) were female and 69% (n=61) completed week 24 with no differences in drop-out by condition (χ2(1,87)=0.70, P=0.490)."<br>...<br>"Of 61 completers, 53% (n=32) remained within 2% of their starting weight. Significant increases occurred over 24 weeks for satisfaction with life and weight-loss self-efficacy regardless of app condition. Diet and physical activity behaviors did not vary by app or week. Negative binomial models indicated that those receiving the full app remained active users of the app for 46 days longer than controls (P=0.017). Users of the full version of the app also reported that they felt more supported than those with the control app (P=0.006)."		
**1b-v) CONCLUSIONS/DISCUSSION in abstract for negative trials**		
While some aspects of the intervention app such as usage and user feedback showed promise, there were few observable effects on behavioral and psychological outcomes. Future evaluation of the app may need to implement more progressive research methods or target more specific populations in order to better understand the utility of the coping interface."		
INTRODUCTION		
**2a-i) Problem and the type of system/solution**		
"According to the World Health Organisation, 1.9 billion adults were overweight or obese in 2016 [1]. In response to the challenge of weight management, many weight loss programs have been developed. Despite the fact that many people have initial success in changing their dietary and/or physical activity behaviors to lose weight, few successfully maintain their lost weight over the longer term [2]. For example, only 20% of people from the National Weight Control Registry in the US managed to maintain initial weight losses after 2 years [3]. Successfully maintaining weight loss for 2-5 years greatly increases the likelihood of longer term success [4], as does increasing the duration of exposure to the weight loss program [5]. However, it currently appears as though weight loss is regained in a linear fashion with few mitigating factors [6]."<br>...<br>"Recent technological progress has resulted in a shift from web-based to smart phone-based weight management interventions, with or without face-to-face support with some promising results [9, 10]. Mobile phones could be used to extend the active duration of engagement with a weight management program, even via simple features as a text message [11]. Therefore, applications (apps) may be a useful delivery mechanism for prolonging weight management attempts and consequently weight-loss maintenance. Digital interventions are often described as more cost-effective and able to be wider-reaching than more intensive face-to-face programs. As technology becomes more sophisticated, the ability to provide just-in-time intervention means that portable devices may also be able to provide intervention at critical times."<br>...<br>"Therefore, the aim of the current study was to develop and test a theoretically and evidence-based mobile phone intervention for weight loss maintenance"<br>...		
**2a-ii) Scientific background, rationale: What is known about the (type of) system**		
"Recent technological progress has resulted in a shift from web-based to smart phone-based weight management interventions, with or without face-to-face support with some promising results [9, 10]. Mobile phones could be used to extend the active duration of engagement with a weight management program, even via simple features as a text message [11]. Therefore, applications (apps) may be a useful delivery mechanism for prolonging weight management attempts and consequently weight-loss maintenance."<br>...<br>"a review of just-in-time interventions suggested that portable devices may be useful to enhance cognitive-behavioral therapy for weight loss programs [12]. Mobile phones also provide an avenue for regular self-monitoring which have been strongly linked with successful behavior change, particularly in weight management [13, 14]. "<br>...<br>"Previous authors have emphasised the importance of theory-based interventions that use scientific evidence and utilise the functionality of modern phones [23, 24, 25]."		
**Does your paper address CONSORT subitem 2b?**		
"Specifically, we aimed to design and evaluate an app to improve psychological wellbeing, engagement with the intervention and, ultimately, weight maintenance outcomes."		
METHODS		
**3a) CONSORT: Description of trial design (such as parallel, factorial) including allocation ratio**		
Figure 1		
**3b) CONSORT: Important changes to methods after trial commencement (such as eligibility criteria), with reasons**		
None of the research methods were changed.		
**3b-i) Bug fixes, Downtimes, Content Changes**		
"Due to technical issues with the app, an update was released after the first week of the trial. This affected iPhone users only. The update was released within two days of a reported fault. Four participants reported technical issues with their app. Two of these (did not see a weekly report and last data entered not saved) resolved themselves and may have been related to a temporary outage of the external database. The other two reports related to the app opening slowly and were both for Android systems. The developers could not replicate this issue and participants persisted with the app despite this inconvenience."		
**4a) CONSORT: Eligibility criteria for participants**		
"Participants had to meet the following eligibility criteria: Adult (18 years or older); lost at least 5% of their body weight within last 2 years; access to bathroom scales; want to continue or maintain their weight loss; own a Smartphone with an operating system appropriate for the app (iPhone or Android); willing to attend a clinic in the central business district 5 times over 6 months."		
**4a-i) Computer / Internet literacy**		
Not relevant. We were recruiting people who owned their own smartphones and therefore would have a baseline level of literacy		
**4a-ii) Open vs. closed, web-based vs. face-to-face assessments:**		
"The primary method of recruitment was through an existing clinical research unit database owned by CSIRO which included the contact details of people who had consented to be contacted about future research. This method was supplemented by local print advertising, promotional news stories, and unaddressed promotional pamphlets delivered by Australia Post. In final recruitment efforts, an external recruitment company was engaged"		
**4a-iii) Information giving during recruitment**		
"After being screened over the phone by the clinical trial manager, potential participants attended a study information session delivered by the principal investigator, received an information sheet and then provided written consent to participate."		
**4b) CONSORT: Settings and locations where the data were collected**		
"Between late 2014 and mid-2015, participants made five visits to the clinical research unit in Adelaide, South Australia. Visits occurred at baseline (week 0), 4 weeks, 8 weeks, 12 weeks and 24 weeks (Figure 1). "		
**4b-i) Report if outcomes were (self-)assessed through online questionnaires**		

"At all visits the clinical trial manager recorded body weight and participants then completed a computer-delivered survey that was programmed in Survey Monkey. These visits generally took less than 15 minutes each."		
**4b-ii) Report how institutional affiliations are displayed**		
Participants attended the CSIRO clinic, it is branded as such (not reported specifically in paper)		
**5) CONSORT: Describe the interventions for each group with sufficient details to allow replication, including how and when they were actually administered**		
**5-i) Mention names, credential, affiliations of the developers, sponsors, and owners**		
"Both groups received a smartphone app designed by the research team to be used without any additional face-to-face support. The app was developed by an external company (Enabled, Adelaide, South Australia) with close oversight from the study team and programmed for both iPhone and Android users."		
**5-ii) Describe the history/development process**		
"The development and features of the full version of the MotiMate app is described in detail elsewhere [29]."		
**5-iii) Revisions and updating**		
"Content did not change throughout the trial."		
**5-iv) Quality assurance methods**		
Not relevant, objective interractions captured,		
**5-v) Ensure replicability by publishing the source code, and/or providing screenshots/screen-capture video, and/or providing flowcharts of the algorithms used**		
Potential commericialisation, therefore source code not provided. Logic is described in previous paper.		
**5-vi) Digital preservation**		
Screenshots included in Figure 2		
**5-vii) Access**		
"At the baseline visit, the intervention or control app was manually loaded onto the participants' phones."		
**5-viii) Mode of delivery, features/functionalities/components of the intervention and comparator, and the theoretical framework**		
Described in detail in other paper. Summarized in Table 1		
**5-ix) Describe use parameters**		
"Briefly, both study apps had the same visual appearance, labelled MotiMate and designed for daily use."		
**5-x) Clarify the level of human involvement**		
"Clinic staff confirmed correct allocation and recorded allocation in the participant clinic record. They then showed the participant the app icon and ensured that participants could log in to the app using the account credentials entered at set-up. To replicate a 'real world' setting where the app would be downloaded from an app store, clinic staff did not provide an overview of the app to participants."		
**5-xi) Report any prompts/reminders used**		
described in Table 1		
**5-xii) Describe any co-interventions (incl. training/support)**		
Not relevant, none included.		
**6a) CONSORT: Completely defined pre-specified primary and secondary outcome measures, including how and when they were assessed**		
See descriptions starting from page 10 under Primary Study Outcomes		
**6a-i) Online questionnaires: describe if they were validated for online use and apply CHERRIES items to describe how the questionnaires were designed/deployed**		
Surveys completed in the clinic on computers - not online.		
**6a-ii) Describe whether and how "use" (including intensity of use/dosage) was defined/measured/monitored**		
"Interactions with the app including logging in and accessing each of the core features were captured by the app and sent to an external database."<br>...		
**6a-iii) Describe whether, how, and when qualitative feedback from participants was obtained**		
All qualitative with some open-ended items		
**6b) CONSORT: Any changes to trial outcomes after the trial commenced, with reasons**		
"Between late 2014 and mid-2015, participants made five visits to the clinical research unit in Adelaide, South Australia. Visits occurred at baseline (week 0), 4 weeks, 8 weeks, 12 weeks and 24 weeks (Figure 1). "		
**7a) CONSORT: How sample size was determined**		
**7a-i) Describe whether and how expected attrition was taken into account when calculating the sample size**		
"Power calculations were based on changes in mood observed in our previous study [26]. In a sample with 44 females divided into two conditions, we were able to detect a moderate effect (0.45) for changes in mood. The initial aim was to recruit 150 volunteers to allow for 30% drop out [26, 27] and the inclusion of males which may increase the variability in observations."		
**7b) CONSORT: When applicable, explanation of any interim analyses and stopping guidelines**		
See descriptions starting from page 10 under Primary Study Outcomes		
**8a) CONSORT: Method used to generate the random allocation sequence**		
"The clinical trial manager allocated participants based on their ID using a random number generator."		
**8b) CONSORT: Type of randomisation; details of any restriction (such as blocking and block size)**		
"During randomization, subjects were balanced for sex, age and weight status (currently overweight/obese or not)."		
**9) CONSORT: Mechanism used to implement the random allocation sequence (such as sequentially numbered containers), describing any steps taken to conceal the sequence until interventions were assigned**		
"All participants received an app called MotiMate and were blinded regarding their allocation. None of the investigators were involved with participant allocation. Due to the collection of objective usage data (described further below), investigators could not be blinded surrounding participant allocation; some participants had app feature interactions only available on the MotiMate intervention app"		
**10) CONSORT: Who generated the random allocation sequence, who enrolled participants, and who assigned participants to interventions**		
"The clinical trial manager allocated participants based on their ID using a random number generator."		
**11a) CONSORT: Blinding - If done, who was blinded after assignment to interventions (for example, participants, care providers, those assessing outcomes) and how**		
**11a-i) Specify who was blinded, and who wasn't**		
"All participants received an app called MotiMate and were blinded regarding their allocation. None of the investigators were involved with participant allocation. Due to the collection of objective usage data (described further below), investigators could not be blinded surrounding participant allocation; some participants had app feature interactions only available on the MotiMate intervention app"		
**11a-ii) Discuss e.g., whether participants knew which intervention was the "intervention of interest" and which one was the "comparator"**		
""All participants received an app called MotiMate and were blinded regarding their allocation. None of the investigators were involved with participant allocation. Due to the collection of objective usage data (described further below), investigators could not be blinded surrounding participant allocation; some participants had app feature interactions only available on the MotiMate intervention app"		
**11b) CONSORT: If relevant, description of the similarity of interventions**		
Table 1		
**12a) CONSORT: Statistical methods used to compare groups for primary and secondary outcomes**		
"All analyses were performed in SPSS version 20 (IBM, New York, USA). The primary analyses involved intention-to-treat methods using mixed modelling to assess differences in wellbeing, weight, dietary intake and physical activity levels over the study period between the intervention groups.<br>Given the smaller-than-desired final sample, preliminary bivariate correlations were used to assess the relevance of including all confounding variables. Dietary restraint was only weakly associated with a small number of the outcomes and consequently was not controlled for in any of the models. Neuroticism, self-esteem and dispositional optimism (life orientation) related moderately to most of the psychological outcomes. For consistency, these variables were included in models assessing wellbeing, coping, resilience, and self-efficacy. All models also controlled for participant sex and age. The primary dependent variables were app condition, changes over time (by week) and the interaction between these two variables. In the presence of significant interaction effects between app condition and week, pairwise comparisons were made using Bonferroni adjustments.<br>Due to skew in the app interaction data, comparisons of usage of features were made using negative binomial linear models. These models were over-dispersed, so the parameter model was estimated by SPSS rather than set to 1. App condition was compared controlling for sex and age in these models."		
**12a-i) Imputation techniques to deal with attrition / missing values**		
"The primary analyses involved intention-to-treat methods using mixed modelling to assess differences in wellbeing, weight, dietary intake and physical activity levels over the study period between the intervention groups. "		
**12b) CONSORT: Methods for additional analyses, such as subgroup analyses and adjusted analyses**		
No subgroup analyses		
RESULTS		
**13a) CONSORT: For each group, the numbers of participants who were randomly assigned, received intended treatment, and were analysed for the primary outcome**		
Figure 3		
**13b) CONSORT: For each group, losses and exclusions after randomisation, together with reasons**		

Figure 3		
**13b-i) Attrition diagram**		
Figure 5		
**14a) CONSORT: Dates defining the periods of recruitment and follow-up**		
"Between late 2014 and mid-2015, participants made five visits to the clinical research unit in Adelaide, South Australia."		
**14a-i) Indicate if critical “secular events” fell into the study period**		
No changes		
**14b) CONSORT: Why the trial ended or was stopped (early)**		
Not stopped early		
**15) CONSORT: A table showing baseline demographic and clinical characteristics for each group**		
Reported in text due to high number of tables.<br>"The sample was between the ages of 20 and 67 years old and mostly female (n=66; 75%). A majority owned an iPhone (n=62; 70.5%) rather than an Android handset. The group’s starting weight ranged from 53.4 to 170.4kg with an average of 85.8kg (SD=22.08). Body Mass Index was between 20.9 and 60.8 with 19.3% (n=17), 39.8% (n=35), 23.9% (n=21) and 17.0% (n=15) of the sample being normal weight, overweight, obese (category I) and obese (category II) respectively."		
**15-i) Report demographics associated with digital divide issues**		
"The sample was between the ages of 20 and 67 years old and mostly female (n=66; 75%). A majority owned an iPhone (n=62; 70.5%) rather than an Android handset. The group’s starting weight ranged from 53.4 to 170.4kg with an average of 85.8kg (SD=22.08). Body Mass Index was between 20.9 and 60.8 with 19.3% (n=17), 39.8% (n=35), 23.9% (n=21) and 17.0% (n=15) of the sample being normal weight, overweight, obese (category I) and obese (category II) respectively. "		
**16a) CONSORT: For each group, number of participants (denominator) included in each analysis and whether the analysis was by original assigned groups**		
**16-i) Report multiple “denominators” and provide definitions**		
Reported throughout results		
**16-ii) Primary analysis should be intent-to-treat**		
Analysis was intention-to-treat		
**17a) CONSORT: For each primary and secondary outcome, results for each group, and the estimated effect size and its precision (such as 95% confidence interval)**		
Reported throughout results section.		
**17a-i) Presentation of process outcomes such as metrics of use and intensity of use**		
Figure 5		
**17b) CONSORT: For binary outcomes, presentation of both absolute and relative effect sizes is recommended**		
Not relevant		
**18) CONSORT: Results of any other analyses performed, including subgroup analyses and adjusted analyses, distinguishing pre-specified from exploratory**		
Not relevant		
**18-i) Subgroup analysis of comparing only users**		
Included in section on app usage		
**19) CONSORT: All important harms or unintended effects in each group**		
Not relevant		
**19-i) Include privacy breaches, technical problems**		
"Technical errors throughout the trial<br>Due to technical issues with the app, an update was released after the first week of the trial. This affected iPhone users only. The update was released within two days of a reported fault. Four participants reported technical issues with their app. Two of these (did not see a weekly report and last data entered not saved) resolved themselves and may have been related to a temporary outage of the external database. The other two reports related to the app opening slowly and were both for Android systems. The developers could not replicate this issue and participants persisted with the app despite this inconvenience."		
**19-ii) Include qualitative feedback from participants or observations from staff/researchers**		
Feedback was collected in quantitative form		
DISCUSSION		
**20) CONSORT: Trial limitations, addressing sources of potential bias, imprecision, multiplicity of analyses**		
**20-i) Typical limitations in ehealth trials**		
"Close to 40% of people continued to lose weight while on this trial even though they had no support specifically directed towards weight loss from the app."<br>....<br>"At the start of our trail participants were within different ranges from their lowest ever weights and they each had different timeframes with which they had been maintaining their weight, as well as different experiences with weight loss programs prior to starting the study. It would have been interesting to explore how these factors may have altered weight outcomes, however our ability to do this was limited due to the sample size. The choice to recruit people with a range of weight management experiences was a purposeful one to assess if the MotiMate design could be effective in a real-world setting where people have had a variety of weight loss experiences; however this may have also added increased variability to the outcomes "		
**21) CONSORT: Generalisability (external validity, applicability) of the trial findings**		
**21-i) Generalizability to other populations**		
"The choice to recruit people with a range of weight management experiences was a purposeful one to assess if the MotiMate design could be effective in a real-world setting where people have had a variety of weight loss experiences; however this may have also added increased variability to the outcomes "<br>...<br>"Engagement with the app features related to mood was low. This is likely to explain the absence of differences between the two groups. Due to the nature of the trial, participants were not informed of mood monitoring features..."<br>...<br>"Trialling the app in an uncontrolled sample would allow us to target a potentially more appropriate market in the future."<br>...<br>"Engagement with the app and intention to continue using fell over 6 months for both apps. Aside from early drops in usage, there was a visible decrease in motivation at week 8. This is an observation that we have made in similar trials [46]. In order to improve the testing of app-based programs in the future, alternative methods of evaluation may be needed including adaptive intervention designs [58]. It is also important to note that while participant may not be recording their behaviors into the app, this does not necessary mean that they are not performed these behaviors. It is likely that there is a point where behaviors such as diet monitoring become habitual and there is no need to rely on tools for assistance."		
**21-ii) Discuss if there were elements in the RCT that would be different in a routine application setting**		
It does not seem of high relevance for discussion given the app was designed to be used in the real world. Only the clinic measurements were different and this is the case with all research which collects data without applying observational methods.		
**22) CONSORT: Interpretation consistent with results, balancing benefits and harms, and considering other relevant evidence**		
**22-i) Restate study questions and summarize the answers suggested by the data, starting with primary outcomes and process outcomes (use)**		
"In this highly controlled, 6-month clinical trial of the MotiMate app we were unable to show any additional benefits of persuasive features and mood monitoring in terms psychological wellbeing and weight maintenance for participants. This is despite observations of longer engagement with the app, more exercise entries and, more positive rating of the intervention app by users. There were improvements in weight-loss self-efficacy and life satisfaction throughout the trial in both groups. These are important constructs for wellbeing and weight maintenance. Interaction effects were observed for anxiety and negative affect. However, post-hoc analyses revealed that these may have been driven by baseline differences and not the intervention per se. A significant interaction effect for changes in resilience was also observed with the intervention group having significant falls in the free-living period while the control group did not. There were minimal differences observed in lifestyle behaviors and other subjective wellbeing constructs"		
**22-ii) Highlight unanswered new questions, suggest future research**		
"Engagement with the app features related to mood was low. This is likely to explain the absence of differences between the two groups. Due to the nature of the trial, participants were not informed of mood monitoring features. Qualitative feedback (not reported) indicated that some people were not receptive to tracking mood. One participant even indicated that they only ever had “one mood” and there was “no need to record it”. Indeed, a review of emotion research suggested individual variability in emotional granularity [47]. Trialling the app in an uncontrolled sample would allow us to target a potentially more appropriate market in the future. "<br>...<br>"Since this study started, recent evidence has emerged that suggests that resource depletion theory may not be as strong as has been previously thought [53]. More recent studies have failed to replicate observations consistent with ego depletion [54, 55] and have called in to question the presence of the described effects. Ego-depletion is a relatively new theory and further studies may be needed to better understand ego depletion and its relationship to eating habits and weight management. Emotion regulation strategies may benefit those prone to emotional eating more observably than other groups."		
Other information		
**23) CONSORT: Registration number and name of trial registry**		
Australian New Zealand Clinical Trials Registry ACTRN12614000474651		
**24) CONSORT: Where the full trial protocol can be accessed, if available**		
Not available		

**25) CONSORT: Sources of funding and other support (such as supply of drugs), role of funders**		
Bupa Health Foundation is a Principle Partner for the development and evaluation of the MotiMate app. They had no direct involvement with the development and evaluation of MotiMate.		
**X26-i) Comment on ethics committee approval**		
"The study was approved by the CSIRO Human Research Ethics Committee (14/02) in April 2014 and registered with the Australian New Zealand Clinical Trials Registry (ACTRN12614000474651)."		
**x26-ii) Outline informed consent procedures**		
"After being screened over the phone by the clinical trial manager, potential participants attended a study information session delivered by the principal investigator, received an information sheet and then provided written consent to participate."		
**X26-iii) Safety and security procedures**		
Not relevant		
**X27-i) State the relation of the study team towards the system being evaluated**		
All government employees		
